# Plasma D-dimer level is associated with clinical outcomes in patients with atrial fibrillation related acute ischemic stroke after pneumonia

**DOI:** 10.1186/s12883-021-02168-x

**Published:** 2021-03-27

**Authors:** Xu Yang, Taoli Lu, Zhanli Qu, Yi Zhang, Pingping Liu, Ying Ma

**Affiliations:** 1 Department of Neurology, Nanchong Central Hospital; The Second Clinical Medical School, North Sichuan Medical College, Nanchong, 637000 Sichuan China; 2 Department of Neurology, Chengdu second people’s hospital, Chengdu, 610015 Sichuan China; 3 Department of Neurology, Affiliated Hospital of North Sichuan Medical College, Nanchong, 637000 Sichuan China

**Keywords:** Acute ischemic stroke, Atrial fibrillation, D-dimer, Pneumonia, Outcome

## Abstract

**Background:**

Pneumonia is related to poor prognosis in acute ischemic stroke (AIS), and its risk might be higher in atrial fibrillation (AF) related AIS with elevated plasma D-dimer. The aim of our study was to investigate the prognostic value of D-dimer for predicting clinical outcome of AF-related AIS with pneumonia.

**Method:**

AF-related AIS patients with pneumonia were prospectively enrolled. Receiver operating characteristic (ROC) curve was used to determine the optimal D-dimer point for 3-month mortality and death/severe disability. The associations between the D-dimer and 3-month mortality and death/severe disability were assessed by multivariable logistic regression analysis.

**Results:**

A total of 415 patients were enrolled in this study. ROC curve analysis showed that the optimal cut point of D-dimer for 3-month death/severe disability and mortality were D-dimer≥2.35 mg/l and D-dimer≥3.35 mg/l, respectively. Multivariable logistic regression analysis showed that D-dimer≥2.35 mg/l [adjusted odds ratio (aOR) 5.99, 95% confidence interval (CI): 3.04–11.83, P<0.001], higher NIHISS score (aOR:1.53, 95% CI: 1.38–1.69, P<0.001) and larger infarct volume (aOR 1.01, 95% CI: 1.01–1.02, P<0.001) were associated with increased risk of 3-month death/severe disability), and anticoagulant was associated with decreased risk of death/severe disability (aOR:0.21, 95% CI: 0.09–0.47, P<0.001). Higher NIHISS score (aOR:1.64, 95% CI: 1.38–1.94, P<0.001), older age (aOR 1.08, 95% CI: 1.02–1.14, *P* = 0.007), D-dimer≥3.35 mg/l (OR 8.49, 95% CI: 4.13–17.84,P<0.001), larger infarct volume (aOR 1.02, 95% CI: 1.00–1.03, *P* = 0.014), and higher CRUB-65 score (aOR 6.43, 95% CI: 3.10–13.34, P<0.001) were associated with increased risk of 3-month mortality.

**Conclusions:**

AF-related AIS patients with concurrent high D-dimer and pneumonia increased risk of 3-month mortality and death/severe disability, plasma D-dimer may have predictive value in outcome after AF-related AIS with pneumonia.

## Background

Atrial fibrillation (AF) is the most common arrhythmia, which associated with increased risk of mortality and disability after stroke. The large infarcts characteristically associated with AF reflect the larger size of stasis thrombi that form in the low shear environment of the left atrium [1, 2]. 30-day mortality of AF-related stroke was more than 24%, another 35% patients had severe disability [3–5].

D-dimer is a protein produced by the degradation of cross-linked fibrin by factor XIII. It is used as a screening tool for venous thromboembolism, but its role in other pathologies has not been fully elucidated [6]. AF leads to decreased flow in the left atrial appendage and the atrium, which combine with platelet activation and endothelial dysfunction, increase coagulation activity and lead to stroke. Studies had showed that patients with AF could increase D-dimer levels [7–9], and higher D-dimer levels could increase risk of future cerebrovascular and cardiovascular events [10–12], increased D-dimer levels had been reported to be proportional to the severity of stroke. In addition, pneumonia leads to fibrin deposition in the alveoli and interstitial lungs, inducing activation of the intravascular and extravascular coagulation systems [13]. D-dimer may reflect the effect of infection on coagulation. Some studies had reported that elevated D-dimer levels in pneumonia patients might indicate hypercoagulability and increase the risk of thrombosis [14–16]. Pneumonia is the most common complication of AIS, which could lead to poor clinical outcomes and increase mortality, previous study had showed a significant correlation between D-dimer levels and pneumonia severity, and higher D-dimer levels in patients with pneumonia could identify individuals at increased risk for in-hospital death [17]. Studies had showed that elevated D-Dimer was associated with major complications during hospitalization in patients with pneumonia [18, 19].

The predictive value of D-dimer in clinical outcome among AF-related AIS with pneumonia has not been elucidated. Therefore, in the present study, we investigated the prognostic value of D-dimer for predicting clinical outcome of AF-related AIS with pneumonia.

## Methods

### Study population

This study was a multicenter prospective study conducted in three medical centers: Nanchong Central Hospital of the Second Clinical Medical School, North Sichuan Medical College, the Chengdu second people’s hospital, and affiliated Hospital of North Sichuan Medical College. Patients with AF-related AIS were admitted within 7 days to the stroke ward between July 2015 and March 2019. AIS is defined as sudden loss of neurological function with symptoms lasting for more than 24 h. Brain CT scan was normal or acute ischemic changes, diffusion-weighted imaging showed acute ischemic changes. TOAST classification was used for determining stroke subtype. AF-relate stroke was confirmed by an experienced neurologist who blinded to the study. The severity of stroke was assessed using the National Institutes of Health Stroke Scale (NIHSS) [20].

Pneumonia was diagnosed based on clinical history and symptoms (cough, fever, purulent secretions), and laboratory parameters of respiratory tract infection, and was confirmed by chest CT.

Only patients who met all the following criteria were included in the study: 1. First new-onset AF-related AIS within 7 days. 2. AIS was consistent with clinical manifestations. 3. Patients had pneumonia within 7 days after stroke. 4. Patients have electrocardiogram-confirmed AF within the prior 6 months. All patients received standard treatment, including anticoagulant treatment or antiplatelet treatment, lipid-lowering treatment, and rehabilitation. Exclusion criteria**:** 1. Cerebral hemorrhage; 2. Renal failure (glomerular filtration rate < 30 ml/min.1.73m^2^); 3. Active malignancies; 4. Aortic dissection.5. Presence of thrombus in the left atrium/appendage.

### Data collection

Plasma D-dimer levels were measured with an enzyme-linked fluorescent immunoassay method (Lituo Biotechnology Co. Ltd., Hunan, China) in all patients on admission. Other laboratory data on admission [white blood cells, hemoglobin, liver function, kidney function, LDL-cholesterol, serum glucose, glycated hemoglobin, brain natriuretic peptide, and procalcitonin (PCT)] were performed. Body temperature was checked at least three times a day. In case of suspect clinical infection, chest CT, PCT, blood and sputum cultivations were rechecked. The severity of pneumonia was assessed using CRUB-65 score within 48 h after pneumonia. The CURB-65 index uses five core clinical features: new onset confusion, urea > 7 mmol/l, respiratory rate 30 breaths/min, systolic blood pressure [21]. Infarct volume was manually calculated by radiologist using hand tracing method in terms of brain CT.

All patients were followed up for 3 months. Clinical outcome was defined as 3-month mortality (include the in-hospital mortality) and death/ severe disability (scores 3–5 on the modified Rankin Scale [mRS]) after AF related AIS, good outcome was defined as mRS ≤ 2 [22].

### Statistical analysis

All patients were divided death/severe disability and good outcome groups, and then, patients were classified into death and survivor groups. To identify differences between subgroups, the Pearson χ^2^ test was used for categorical variables. Distributions of continuous variables were determined by the Kolmogorov–Smirnov test, while Mann–Whitney two sample test was applied in case of non-normal distributions. Receiver operating characteristic (ROC) curve analysis was used to evaluate sensitivity, specificity and to determine the optimal cut point of D-dimer for 3-month mortality and death/ severe disability. We then performed logistic regressions analyses to determine the association between D-dimer and 3-month mortality and death/ severe disability. Results were expressed as adjusted odds ratios (aOR) with the corresponding 95% confidence interval (CI). SPSS 22.0 statistical software was used for data analyses. *P* < 0.05 was used as a significance level.

## Results

### Patient characteristics

415 AF-related AIS patients with pneumonia were included in the study, 211 patients were female (52.45%) and 184 patients were male (47.55%), with an average age of 67.62 ± 9.84 years old (ranging from 40 to 93 years old), median NIHSS score was 11(7–14), medians international standard ratio (INR) was 1.36(1.09–1.95). 19.76% (82) patients were treated with anticoagulation, medians D-dimer value was 1.9 mg/l (1.2–3.0 mg/l). There were 123(29.64%) cases of diabetes, 265(63.86%) cases of hypertension, 278 (66.99%) cases of hyperlipidemia, 126(30.36%) cases of smoking and 147(35.42%) cases of drinking. During 3 months of follow-up, 50 (12.05%)patients had died (17 patients had died during hospitalization), 178 (42.89%) patients had severe disability (Table 1).

**Table 1:** The baseline characteristics between good outcome and death/severe disability groups

	Good outcome group (187)	Death/severe disability group (228)	OR(95%CI)	P*
Age, y (Mean SD)	66.32 ± 9.97	68.68 ± 9.63		**0.005**
NIHSS score, median (IQR)	7(5–11)	13(10–16)		**<0.001**
D-dimer, median (IQR)	1.30(0.80–2.00)	2.50(1.60–3.88)		**<0.001**
INR, median (IQR)	1.34(1.06–1.97)	1.37(1.10–1.93)		**0.838**
CHA2DS2-VASc, median (IQR)	4.00(4–5)	4.0(4–5)		**0.360**
Infarct volume, ml, median (IQR)	51(29–76)	81(50–127)		**<0.001**
CURB-65 score, median (IQR)	2(1–2)	2(1–3)		**0.005**
Females, n(%)	88(47.06)	123(53.95)	1.32 (0.89–1.94)	0.163
Male, n(%)	99(52.94)	105(46.05)	1.32 (0.89–1.94)	0.163
BMI ≥ 24 kg/m, n(%)	55(29.41)	68(29.82)	1.02(0.67–1.56)	0.927
Hypertension, n(%)	120(64.17)	145(63.60)	0.98(0.65–1.46)	0.904
Current Smoking, n(%)	51(27.27)	75(32.89)	1.31(0.86–2.00)	0.215
Current alcohol drinking,n(%)	66(35.29)	81(35.53)	1.01(0.67–1.51)	0.961
Diabetes, n(%)	58(31.01)	65(28.51)	0.89(0.58–1.35)	0.578
Hyperlipidemia, n(%)	121(64.71)	157(68.86)	1.21(0.80–1.82)	0.371
Family history of stroke, n(%)	46(24.60)	48(21.05)	0.82(0.52–1.30)	0.390
Thrombolytic therapy, n(%)	26(13.90)	25(10.96)	0.76(0.42–1.37)-	0.763
Thrombectomy, n(%)	10(5.35)	13(5.70)	1.07(0.46–2.50)	0.875
Thrombolytic therapy+ Thrombectomy, n(%)	4(2.14)	4(1.75)	0.82(0.20–3.31)	0.777
Medications use				
Antihypertensive, n(%)	96(51.34)	133(58.33)	1.38(0.90–1.96)	0.154
Anticoagulant, n(%)	61(32.62)	21(9.21)	0.21(0.12–0.36)	**0.000**
Antiplatelets, n(%)	54(28.88)	55(24.12)	0.78(0.51–1.21)	0.274
Lipid-lowering medications, n(%)	109(58.29)	132(57.89)	0.98(0.67–1.46)	0.935

Bold indicates *P*-values less than 0.05.

*Comparison between good outcome and death/severe disability groups. The data are presented as median values (interquartile range [IQR]), numbers (%), or mean values (±standard deviation). Categorical variables are expressed as frequency (percent) for *P* values. Baseline characteristics were compared between the 2 subgroups by univariate analysis using Pearson χ2, distributions of continuous variables were determined by the Kolmogorov–Smirnov test, Mann–Whitney two sample test was applied in case of non-normal distributions

### Factors related to death/severe disability

228 (228/415,54.94%) patients had death/severe disability after 3 months of follow-up. The patients with death/severe disability had older age (68.68 ± 9.63 vs 66.32 ± 9.97,*P* = 0.005), higher NIHSS score [13(10–16) vs 7(5–11), P<0.001], higher D-dimer levels [2.50(1.60–3.88) vs 1.30(0.80–2.00), P<0.001], larger infarct volume [81(50–127) vs 51(29–76), P<0.001], and higher CRUB-65 score [2(1–3) vs 2(1–2), P = 0.005], lower percentage of anticoagulant (9.21% vs 32.62%, P<0.001) than those who good outcome (Table 1).

ROC curve showed that D-dimer predict death/ severe disability with AUC of 0.78 (95%CI 0.73 to 0.82). The optimal cut point off D-dimer for death/ severe disability was D-dimer≥2.35 mg/l. Using D-dimer ≥2.35 mg/l to predict death/severe disability, the sensitivity was 55.70%, the specificity was 83.96%, positive predictive value was 80.89%; and negative predictive value was 60.85%.

In the multivariate logistic regression model, after adjusting for all the confounding factors (Model 1), higher NIHISS score, higher D-dimer value and larger infarction volume were associated with increased risk of 3-month death/severe disability (aOR:1.50, 95% CI: 1.36–1.67, P<0.001; aOR 2.29, 95% CI: 1.70–3.08, P<0.001; aOR 1.01, 95% CI: 1.01–1.02, P<0.001;respectively),and anticoagulant was associated with decreased risk of 3-month death/severe disability (aOR:0.22, 95% CI: 0.10–0.49, P<0.001); when D-dimer≥2.35 mg/l was entered into multivariate logistic regression (Model 2), higher NIHISS score, D-dimer≥2.35 mg/l and larger infarction volume were associated with increased risk of 3-month death/severe disability (aOR:1.53, 95% CI: 1.38–1.69, P<0.001; aOR 5.99, 95% CI: 3.04–11.83, P<0.001; aOR 1.01, 95% CI: 1.01–1.02, P<0.001respectively) (Table 2),and anticoagulant was associated with decreased risk of death/severe disability (aOR:0.21, 95% CI: 0.09–0.47, P<0.001) (Table 2), the CURB-65 score was not associated with the risk of death/severe disability(*P* ≥ 0.05).

**Table 2:** Logistic regression analysis of the risk factors associated with Death/severe disability

	aOR (95% CI)	P*
Model 1(D-dimer value)		
NIHSS score	1.50(1.36–1.67)	**<0.001**
D-dimer value	2.29(1.70–3.08)	**<0.001**
Anticoagulant	0.22(0.10–0.49)	**<0.001**
Infarct volume	1.01(1.01–1.02)	**<0.001**
CURB-65 score	1.30(0.98–1.73)	0.071
Model 2(D-dimer≥2.35 mg/l)		
NIHSS score	1.53(1.38–1.69)	**<0.001**
D-dimer≥2.35 mg/l	5.99(3.04–11.83)	**<0.001**
Anticoagulant	0.21(0.09–0.47)	**<0.001**
Infarct volume	1.01(1.01–1.02)	**<0.001**
CURB-65	1.33(1.00–1.76)	0.05

Bold indicates P-values less than 0.05.

*Multivariable adjusted for age, NIHSS score, INR, CHA2DS2-VASc, infarct volume, CRUB-65 score, gender, BMI, hypertension, current smoking, alcohol drinking, diabetes, hyperlipidemia, family history of stroke, thrombolytic therapy, thrombectomy, thrombolytic therapy + thrombectomy, medications use

### Factors related to death

After 3 months of follow-up, 50 (98/387,25.32%) patients died. The patients who died had older age (71.49 ± 10.27 vs 67.09 ± 9.68, *P* = 0.005), higher NIHSS score[18(14–20) vs 10(7–13), *P* < 0.001], higher D-dimer levels [3.85(1.98–4.65) vs 1.80(1.10–2.70),P < 0.001], larger infarct volume [118(68–135) vs 61(34–102), P<0.001], and higher CRUB-65 score [3(2–3) vs 2(1–2), *P* < 0.001] than those who stayed alive(Table 3).

**Table 3:** The baseline characteristics between survivor and death groups

	Survivor group (365)	Death group (50)	OR(95%CI)	P*
Age, y (Mean SD)	67.09 ± 9.68	71.49 ± 10.27		**0.005**
NIHSS score, median (IQR)	10(7–13)	18(14–20)		**<0.001**
D-dimer, median (IQR)	1.80(1.10–2.70)	3.85(1.98–4.65)		**<0.001**
INR, median (IQR)	1.35(1.09–1.93)	1.40(1.13–2.03)		**0.722**
CHA2DS2-VASc, median (IQR)	4.00(4–5)	4.0(4–5)		**0.676**
Infarct volume, ml, median (IQR)	61(34.5–102.5)	118(60.8–135.3)		**<0.001**
CURB-65 score, median (IQR)	2(1–2)	3(2–3)		**<0.001**
Females, n(%)	187(51.23)	24(48.00)	0.88 (0.49–1.59)	0.668
Male, n(%)	178(48.77)	26(52.00)	0.88 (0.49–1.59)	0.668
BMI ≥ 24 kg/m, n(%)	110(30.14)	13(26.00)	0.81(0.42–1.59)	0.548
Hypertension, n(%)	233(63.84)	32(64.00)	1.01(0.54–1.86)	0.982
Current Smoking, n(%)	106(29.04)	20(40.00)	1.63(0.89–3.00)	0.114
Current alcohol drinking, n(%)	124 (33.97)	23(46.00)	1.66(0.91–3.01)	0.095
Diabetes, n(%)	112(30.68)	11(22.00)	0.64(0.32–1.29)	0.207
Hyperlipidemia, n(%)	147(40.27)	31(62.00)	0.78(0.42–1.44)	0.424
Family history of stroke, n(%)	86(23.56)	8(16.00)	0.62(0.28–1.37)	0.231
Thrombolytic therapy, n(%)	43(11.78)	816.00)	1.43(0.63–3.24)	0.394
Thrombectomy, n(%)	18(4.93)	5(10.00)	2.14(0.76–6.05)	0.142
Thrombolytic therapy+ Thrombectomy, n(%)	7(1.92)	1(2.00)	1.04(0.13–8.67)	0.968
Medications use				
Antiplatelets, n(%)	96(26.30)	13(26.00)	0.99(0.50–1.93)	0.964
Lipid-lowering medications, n(%)	213(58.36)	28(56.00)	0.91(0.50–1.65)	0.752

Bold indicates P-values less than 0.05.

*Comparison between survivor and death groups. The data are presented as median values (interquartile range [IQR]), numbers (%), or mean values (±standard deviation). Categorical variables are expressed as frequency (percent) for P values. Baseline characteristics were compared between the 2 subgroups by univariate analysis using Pearson χ2, distributions of continuous variables were determined by the Kolmogorov–Smirnov test, Mann–Whitney two sample test was applied in case of non-normal distributions

17 patients died during hospitalization, the patients who died during hospitalization had higher NIHSS score[19(13–21) vs 10(7–14), P < 0.001], higher D-dimer levels [3.40(1.90–4.40) vs 1.85(1.18–2.93),*P* = 0.002],and higher CRUB-65 score [3 (2–3) vs 2(1–2),P < 0.001] than those non- in-hospital death group.

ROC curve showed high accuracy for D-dimer predict 3-month mortality with AUC of 0.76(95%CI 0.69 to 0.84). The optimal cut point of D-dimer for 3-month mortality was D-dimer≥3.35 mg/l. Using a cutoff point of D-dimer≥3.35 mg/l to predict 3-month mortality, the specificity was 84.93%, positive predictive value was 35.29%; and negative predictive value was 93.94%.

In the multivariate logistic regression model, after adjusting for all the confounding factors (Model 1), higher NIHISS score, higher age, higher D-dimer value, larger infarct volume, and higher CURB-65 score were independently associated with increased risk of 3-month mortality (aOR:1.58, 95% CI: 1.35–1.86, P<0.001; aOR 1.07, 95% CI: 1.01–1.13, *P* = 0.016; aOR 170, 95% CI: 1.27–2.28, *P* < 0.001; aOR 1.01, 95% CI: 1.00–1.03, *P* = 0.020; aOR 6.36, 95% CI: 3.19–12.71, P < 0.001,respectively); when D-dimer≥3.35 mg/l was entered into multivariate logistic regression (Model 2), higher NIHISS score, higher age, D-dimer≥3.35 mg/l, larger infarct volume, and higher CURB-65 score were independently associated with increased risk of 3-month mortality (aOR:1.64, 95% CI: 1. .38–1.94, P<0.001; aOR 1.08, 95% CI: 1.02–1.14, *P* = 0.007; aOR 8.49, 95% CI: 4.13–17.84, *P* < 0.001; aOR 1.02, 95% CI: 1.00–1.03, *P* = 0.014; aOR 6.43, 95% CI: 3.10–13.34, *P* < 0.001, respectively) (Table 4).

**Table 4:** Logistic regression analysis of the risk factors associated with mortality

	aOR (95% CI)	P*
Model1(D-dimer value)		
NIHSS score	1.58(1.35–1.86)	**<0.001**
Age	1.07(1.01–1.13)	**0.016**
D-dimer value	1.70(1.27–2.28)	**<0.001**
Infarct volume	1.01(1.00–1.03)	**0.020**
CURB-65 score	6.36(3.19–12.71)	**<0.001**
Model 2(D-dimer≥3.35 mg/l)		
NIHSS score	1.64(1.38–1.94)	**<0.001**
Age	1.08(1.02–1.14)	**0.007**
D-dimer≥3.35 mg/l	8.49(4.13–17.84)	**<0.001**
Infarct volume	1.02(1.00–1.03)	**0.014**
CURB-65	6.43(3.10–13.34)	**<0.001**

Bold indicates P-values less than 0.05.

*Multivariable adjusted for age, NIHSS score, INR, CHA2DS2-VASc, infarct volume, CRUB-65 score, gender, BMI, hypertension, current smoking, alcohol drinking, diabetes, hyperlipidemia, family history of stroke, thrombolytic therapy, thrombectomy, thrombolytic therapy+ thrombectomy, medications use

In the multivariate logistic regression model, after adjusting for all the confounding factors, higher NIHSS and higher CURB-65 were independently associated with increased risk of in-hospital mortality (aOR 1.55, 95% CI: 1.27–1.90, P < 0.001; aOR 3.73, 95% CI: 1.53–9.11, P < 0.001, respectively);

## Discussion

AF is the most prevalent sustained cardiac rhythm disorder seen in clinical practice. AF is linked to a 5-fold increased risk of cerebrovascular events, and approximately 20% of strokes are related to AF.AF-related strokes impart worse prognosis than those occurring in the absence of AF. The 30-day mortality of AF-related stroke was 24%, another 35% of patients were unable to live independently [2, 4]. In this study, we found that the 3-month mortality was 12.05%, the remaining 365 survivors, severe disability rates were 48.76% (178/365). The mortality and disability rate in this study were lower than those reported in previous studies, which may be due to most of the patients from the level of first-class comprehensive hospital, medical conditions are relatively good. We identified risk factors associated with outcome in AF-related stroke patients with pneumonia, the results showed that the patients with death/severe disability had older age, higher NIHSS score, higher D-dimer levels, larger infarct volume than those with good outcome.

D-dimer is a specific cross-linked fibrin degradation product, it is a sensitive biomarker for indicating coagulation and fibrinolytic activation, which remains stable over time in the absence of any adverse events [23]. D-dimer levels are commonly used to screen for pulmonary embolism and aortic dissection [6, 8]. Previous studies had showed that the level of D-dimer increased after AIS, which might be related to stroke subtype and stroke volume, and significantly increased in cardiogenic embolic ischemic stroke [24, 25]. In recent years, some studies had showed that D-dimer levels could be a valuable and independent short-term prognostic marker for acute stroke [26]. In AF-related AIS patients with pneumonia, the prognostic value of D-dimer levels remains unclear. In our study, we found D-dimer level was significantly elevated (2.24 ± 1.54 mg/l), we have obtained the correlation between D-dimer levels and outcome, the optimal cut point off D-dimer for 3-month death/severe disability and mortality was D-dimer≥2.35 mg/l and ≥ 3.35 mg/l, respectively, after adjusting for fully confounders, we found a significant association of D-dimer≥2.35 mg/l and D-dimer≥3.35 mg/l with increased risks of 3-month death/severe disability and mortality after AF-related AIS with pneumonia. From our results, we could confirm that D-dimer was a valuable prognostic marker of functional outcome and death among AF-related AIS with pneumonia.

The mechanism of the relationship between D-dimer levels and the prognosis of AF-related AIS patients with pneumonia remains unclear. D-dimer levels may reflect ongoing thrombus formation within cerebral vessels or may be a marker of systemic hypercoagulability [27]. The mechanism of high D-dimer levels affected the prognosis of ischemic stroke might be the activation of inflammation through D-dimer. D-dimer can activate monocytes to secrete pro-inflammatory cytokines such as interleukin-6 (IL-6) [28], which play an important role in endothelial dysfunction, atherosclerosis and promotion of hypercoagulability [29, 30]. Elevated D-dimer levels may reflect ongoing cerebrovascular thrombosis by inducing the inflammatory process and may also be a marker of systemic hypercoagulation leading to poor prognosis in patients with acute ischemic stroke [31]. In addition, studies had showed that compared with patients with lacunar stroke or without arterial occlusion, patients with left ventricular venous thrombosis had a higher increased D-dimer levels at the early stage of the disease. Therefore, high D-dimer levels might be a sensitive indicator of poor prognosis, especially in patients with left ventricular venous thrombosis [32]. In addition, Atrial fibrillation is a rapid and uncoordinated atrial activity that results in ineffective atrial contraction, causing blood turbulence in the left atrium and systemically. In fact, compared with laminar or pulsating flowing [33], the loss of shear stress in turbulent conditions is associated with decreased expression of endothelial nitric oxide synthase (eNOS), in addition, patients with atrial fibrillation have a higher degree of systemic inflammation. According to this hypothesis, circulating inflammatory mediators such as TNF-α and IL-18, which are closely associated with endothelial dysfunction and cardiovascular disease [33–35], may induce vascular injury, aggravate the burden of atherosclerosis, and lead to higher cardiovascular morbidity and mortality, as seen in AF subjects [36]. Atrial fibrillation may lead to vascular endothelial dysfunction, on the other hand immunosuppression [37], inhalation of oral secretions, or dysphagia after stroke could increase the risk of pulmonary infection. When patients are in the pulmonary infection state, fibrin deposition in the alveolar compartment enhances the inflammatory response, increases blood flow and vascular permeability, increases neutrophils recruitment and inflammatory mediator production [38]. The synergistic effect of activated inflammation and activated coagulation may lead to adverse outcomes [39]. Thus, D-dimer may represent the biological activation of inflammatory, hemostatic, and fibrinolytic systems.

In our this study, the cut-off value of D-dimer used for predicting death/severe disability was D ≥ 2.35 mg/l, the sensitivity was 55.70%, the specificity was 83.96%, positive predictive value was 80.89%; and negative predictive value was 60.85%. Using a cut-off point of D-dimer≥3.35 mg/l for predicting 3-month mortality, the sensitivity was 60.0%, the specificity was 84.93%, positive predictive value was 35.29%; and negative predictive value was 93.94%. As shown above, poor sensitivity was observed when D-dimer≥3.35 mg/l used for predicting death.

Some limitations of this study are worth considering. First, in this study, we relied on a single baseline D-dimer levels, so we were unable to investigate the association between dynamic changes D-dimer levels and clinical outcomes among AF-related AIS with infection. D-dimer levels should be measured repeatedly for longitudinal analysis, which may provide additional information about development and its prognostic impact. Second, we did not collect the patients’ MRS at discharge, the functional status of the patients at discharge may also affect mortality. Third, left atrial appendage (LAA) is an important source of thrombi in patients with AF, but we lack data of left atrial. Fourth, due to contraindications, some patients were treated with platelet aggregation instead of anticoagulant after stroke, and we did not further analyze these patients, which might have an impact on the study results. The strength of our study was that it was related to the large number of patients, moreover, it was a multicenter clinical prospective observational study with robust and of high clinical relevance results.

## Conclusion

In conclusion, our study showed a positive correlation between increased D-dimer level and unfavorable outcome after AF-related AIS with infection. Our findings substantiated the importance of an early diagnosis of elevated D-dimer in AF-related stroke survivors with infection given the elevated risk of unfavorable outcome. D-dimer levels may have potential predictive value in risk stratification of ischemic stroke.

## Data Availability

Data used in this study may be available by request to corresponding author via email: 2399548051@qq.com

## Abbreviations

AF: Atrial fibrillation
AIS: Acute ischemic stroke
ROC: Receiver operating characteristic
aOR: adjusted odds ratio
NIHSS: National Institutes of Health Stroke Scale
TOAST: Trial of Org 10,172 in Acute Stroke Treatment
mRS: modified Rankin Scale
PCT: Procalcitonin
IQR: Interquartile range
